# Magnetic nanoparticles in square-wave fields for breakthrough performance in hyperthermia and magnetic particle imaging

**DOI:** 10.1038/s41598-024-61580-8

**Published:** 2024-05-10

**Authors:** Gabriele Barrera, Paolo Allia, Paola Tiberto

**Affiliations:** INRiM, Advanced Materials Metrology and Life Sciences, Turin, Italy

**Keywords:** Magnetic properties and materials, Nanoparticles

## Abstract

Driving immobilized, single-domain magnetic nanoparticles at high frequency by square wave fields instead of sinusoidal waveforms leads to qualitative and quantitative improvements in their performance both as point-like heat sources for magnetic hyperthermia and as sensing elements in frequency-resolved techniques such as magnetic particle imaging and magnetic particle spectroscopy. The time evolution and the frequency spectrum of the cyclic magnetization of magnetite nanoparticles with random easy axes are obtained by means of a rate-equation method able to describe time-dependent effects for the particle sizes and frequencies of interest in most applications to biomedicine. In the presence of a high-frequency square-wave field, the rate equations are shown to admit an analytical solution and the periodic magnetization can be therefore described with accuracy, allowing one to single out effects which take place on different timescales. Magnetic hysteresis effects arising from the specific features of the square-wave driving field results in a breakthrough improvement of both the magnetic power released as heat to an environment in magnetic hyperthermia treatments and the magnitude of the third harmonic of the frequency spectrum of the magnetization, which plays a central role in magnetic particle imaging.

## Introduction

The most significant applications of magnetic nanoparticles in present-day personalized nanomedicine are based on the electromagnetic effects of the magnetization reversal under the action of a periodic magnetic field^1–7^. The magnetic moments of nanoparticles of suitable composition and size can be driven by a high-frequency field in such a way that their motion either produces a controllable amount of magnetic energy able to locally heat the environment (resulting in antitumor therapies based on or enhanced by magnetic hyperthermia (MH)^3,8,9^), or generates an induced voltage allowing to trace the position of the nanoparticles themselves within a living environment for diagnostic imaging purposes (magnetic particle imaging (MPI)^5,10,11^). Therapies and diagnostic tools based on the properties of magnetic nanoparticles are particularly looked at because they are repeatable, minimally harmful, basically not invasive and not involving ionizing radiation^3–5,8^. Closely related to MPI, being likewise based on the detection of the harmonics of the magnetization signal, is magnetic particle spectroscopy (MPS), a fast, sensitive laboratory technique presently applied to several bioassays^12–14^.

The electromagnetic effects relevant to therapeutic and diagnostic purposes appear when the nanoparticles are submitted to periodic magnetic fields of sufficiently high frequency (in the 100-300 kHz range for MH, in the 10-30 kHz range for MPI/MPS). The magnetic nanoparticles of choice for biomedical applications are made of iron oxides (IONs) normally in the single-domain condition^15–17^ and most commonly of magnetite (Fe$_3$O$_4$) because of the limited threat posed by this composition to a patient’s health^18,19^ and of the availability of magnetite ferrofluids suitable for *in-vivo* applications^20,21^, together with a sufficiently strong magnetic signal.

In the common practice of both MH and MPI/MPS the ac magnetic fields applied to magnetite nanoparticles are typically characterized by a sinusoidal behaviour. Such a choice is determined by a wide range of reasons, including the easy generation of the signal, the rather simple control electronics, and the ease of interpretation of the magnetic effects.

However, substituting the sinusoidal field waveform with a non-conventional one (such as the trapezoidal wave or the square wave) brings about notable improvements of the efficacy of magnetite nanoparticles in biomedical applications, as put in evidence elsewhere^22^. Recently, magnetic fields characterized by a trapezoidal waveform with almost vertical legs, called almost-square fields, have been generated using a laboratory setup^23^ and experimented on magnetic particles. The results are quite promising in view of the possible application of non-conventional waves as exciting fields for MH^24^. The almost-square signal stays constant for most of the time, periodically switching from a positive value to the one of opposite sign and back in a time significantly shorter than the field’s period, called the inversion time. Such a behaviour markedly affects the one of the magnetization, resulting in favorable effects on the magnetic properties of application-oriented interest in nanomedicine. In particular, a recent systematic *in-vitro* study demonstrates that applying an almost-square field at 300 kHz has considerable capability to increase cancer cell death compared to a sinusoidal treatment of same amplitude, proving that non-harmonic signals are actually able to enhance MHT treatment efficiency against tumor cells^25^.

The present state-of-the-art of the practical realizations of trapezoidal magnetic fields leaves room for a substantial improvement of the waveform thanks to the possible technological evolution of the electronics and the circuitry for generation and control of the magnetic field, with the goal of significantly reducing the inversion time, which is nowadays of the order of the microsecond^23,24^, while keeping an amplitude adequate to the present applications.

As a consequence, it may be useful to study in detail the effects arising when the magnetic nanoparticles are submitted to an ideal square wave (SW) field, viewed as the natural limit of a trapezoidal field wave for vanishingly small inversion time. This is the main task of the work, achieved exploiting a rate-equation treatment of magnetic particles driven by an ideal SW field. In such a case the rate equations admit a simple analytic solution for the magnetization, resulting in a particularly simple and informative description of this quantity and the related effects, both in the time domain and in the frequency domain. The present treatment will highlight the significant advantages of applying to magnetic nanoparticles a SW field instead of a conventional sinusoidal wave of same frequency and amplitude.

## Model

### Rate equations for magnetic nanoparticles

A rate-equation treatment can be applied to determine the time evolution of the magnetization of magnetic nanoparticles characterized by uniaxial effective anisotropy and pictured as double-well systems (DWS) with easy axes randomly pointing in all directions in three dimensions. The magnetic moments on particles are treated in the macrospin approximation (coherent switching)^26^. In the rate-equation framework, changes in the magnetization of the system by effect of magnetic field and/or temperature are ascribed to Néel’s relaxation^27^, so that the method can be applied only when the geometrical axes of particles are fixed in space, a simplifying assumption well satisfied in some important biomedical applications^28^. For nanoparticles immobilized in a tissue or organ, the Brown’s relaxation disappears^29^; moreover, at the frequencies and for the nanoparticle sizes typically exploited in clinical or pre-clinical applications of magnetic hyperthermia^30^ the Brown’s relaxation is expected to be dominated by the much faster Néel’s relaxation even in a fluid environment^29^.

In the present model, the following assumptions concerning the magnetite nanoparticles are made:

- the particles have spherical shape characterized by the diameter *D*;

- they are homogeneously dispersed in a diamagnetic host material (such as a living tissue) without forming chains or aggregates; - their intrinsic magnetic properties are independent of diameter; the magnetization is $M_s=350$ emu/cm$^3$ and the effective magnetic anisotropy is $K_{eff}= 3 \times 10^5$ erg/cm$^3$, values appropriate to single-core nanoparticles of magnetite around room temperature^31–33^. Although both $M_s$ and $K_{eff}$ can be affected by nanoparticle size because of contributions from the particle’s surface^31,33,34^, such an effect is of minor importance in the range of diameters explored in this paper ($D \ge$ 10 nm)^32,33^;

- the particles are considered to be non-interacting; this assumption is supported by the low particle concentration used in clinical applications of magnetic hyperthermia^30^, resulting in a weak dipolar interaction whose effect can be included in the definition of $K_{eff}$^35^.

We consider first the nanoparticles whose easy axis makes an angle $\phi$ with the magnetic field; if their total number (per unit volume) is $N_\phi$, the particles are distributed between the two energy wells according to the occupancy numbers $N_{1\phi }$ and $N_{2\phi } =N_\phi -N_{1\phi }$.

The rate equations determine the time evolution of the reduced occupancy numbers $n_1(t)=N_{1\phi }(t)/N_\phi$ and $n_2(t)=1-n_1(t)$ by effect of the magnetic field and temperature (from now on, the suffix $\phi$ is dropped for the sake of simplicity)^36^. In the case of magnetic DWS, only one independent rate equation is actually needed:

1 $$\begin{aligned} \frac{dn_1}{dt}=-\frac{1}{\tau _1 }n_1+ \frac{1}{\tau _2}n_2 = \frac{1}{\tau _2}- \frac{1}{\tau }n_1 \end{aligned}$$

where

2 $$\begin{aligned} \tau =\frac{\tau _1\tau _2}{\tau _1+\tau _2} \end{aligned}$$

is the effective time constant and the relaxation time for a particle in the *i*th well is:

3 $$\begin{aligned} \tau _i = \tau _0 \exp {\left( \frac{E_{Bi}}{k_B\mathscr {T}}\right) } \hspace{35pt} (i=1,2) \end{aligned}$$

$E_{Bi}$ being the height of the energy barrier as seen from well *i*, $\tau _0$ the inverse of the attempt frequency and $\mathcal {T}$ the absolute temperature. It should be explicitly noted that $E_{Bi}$ and the time constants $\tau _i$ depend on the magnitude of the applied magnetic field and on the angle $\phi$^36^. The effective time constant of the rate-equation method ($\tau$) is of course strictly connected to the Néel’s relaxation time^27^:

4 $$\begin{aligned} \tau _N=\tau _0 \exp {\left( \frac{K_{eff}V}{k_B\mathscr {T}}\right) } \end{aligned}$$

where $V =\pi /6 D^3$ is the volume of the nanoparticle. However, some non-trivial differences between $\tau$ and $\tau _N$ exist. In fact, $\tau _N$ is the relaxation time in the absence of the applied field, i.e., when the two energy wells of the DWS have the same depth. When $H_V=0, \tau =\tau _N/2$ because in this case $\tau _1=\tau _2 =\tau _N$. However, when a magnetic field is applied at a generic angle $\phi$ with respect to the easy axis of the particle, the energy wells are characterized by different barriers $E_{B1},E_{B2}$, the lower energy barrier corresponding to the easy-axis direction at an angle $\phi < \pi /2$ with respect to the magnetic field’s direction (see the sketch reported in panel *b.1* of Fig. 2). Here, the case of collinear particles (i.e., having the same $\phi$ angle) is discussed; the extension to the general case of random easy axes is straightforward.

When *H*(*t*) is an ideal square wave field, the amplitude is always either $H_V$ or $-H_V$, so that its modulus is constant (= $|H_V|$). In this case the energy barriers $E_{Bi}$ ($i=1,2$) as well as the corresponding time constants $\tau _i$ entering the definition of $\tau$ are affected by the field in a rather simple way. In particular, it is shown in the Appendix A that the effective time constant can be written in terms of the Néel’s relaxation time by simply multiplying $\tau _N$ by a field-dependent factor $\beta$:

5 $$\begin{aligned} \tau =\beta (|H_V|) \tau _N = \beta (|H_V|) \, \tau _0 \exp {\left( \frac{K_{eff}V}{k_B\mathscr {T}}\right) }. \end{aligned}$$

As demonstrated in the Appendix A, the dimensionless $\beta (|H_V|)$ function has the form:

6 $$\begin{aligned} \beta (|H_V|) = \frac{\exp \left[ \frac{(E_1+E_2)}{k_B\mathscr {T}}\right] }{\exp \left[ \frac{E_1}{k_B\mathscr {T}} \right] +\exp \left[ \frac{E_2}{k_B\mathscr {T}} \right] } \end{aligned}$$

where $E_1(|H_V|), E_2(|H_V|)$ are the $|H_V|-$dependent energies corresponding to the minima of the two wells. Such a function monotonically decreases with increasing $|H_V|$, starting from 1/2 for $|H_V|=0$. The rate of reduction of $\beta$ as a function of $|H_V|$ significantly depends on the size of nanoparticles; some examples of the $\beta (|H_V|)$ curve are given in the Appendix A for magnetite particles of different diameters. Generally speaking, the relaxation time decreases with increasing the applied field magnitude $|H_V|$, as shown in the Appendix A.

The contribution of the considered subset of particles to the overall time-dependent magnetization of the system along the field’s direction is:

7 $$\begin{aligned} M_{\phi }(t)=M_s (n_1 c_1+ n_2 c_2)=M_s\left[ n_1(c_1-c_2)+c_2 \right] \end{aligned}$$

where $c_i=\cos (\theta _i-\phi )$ ($i=1,2$), $\theta _i$ being the angle of tilt from the easy axis of the magnetic moment of particles by effect of the magnetic field; for a graphic scheme see the Supplementary Information. The subscript $\phi$ is a reminder that the time-dependent quantities $n_1,c_1,c_2$ also depend on $\phi$; $M_s$ is the saturation magnetization of nanoparticles.

Generally speaking, a change of the magnetic field determines a variation of the magnetization $M_\phi (t)$ through two concurring mechanisms: *a*) the redistribution of the population within the two energy wells as a consequence of the change in the energy barrier heights $E_{Bi}$ and *b*) the variation of the angles of tilt $\theta _i$ and consequently of the angular parameters $c_i$. In the general case, both a numeric solution of the rate equation for $n_1(t)$ and a numeric evaluation of the $c_i$ parameters are needed to find $M_\phi (t)$^36^.

In this paper, the amplitude of the SW magnetic field is always the same, $H_V=100$ Oe, as typically found in biomedical applications^37^.

Advantages and limits of rate equations applied to magnetic nanoparticles were discussed in detail elsewhere^22^. A brief summary of the model’s features is given here:

- rate equations are a flexible, effective way to study magnetic hysteresis loops of a DWS assembly submitted to any time-dependent magnetic field;

- the rate-equation treatment of the dynamic behavior of a DWS assembly has the notable advantage of providing an accurate picture of the evolving magnetization without requiring much computational power and time as in numerical treatments involving the solution of stochastic Landau-Lifshitz (LL) or Landau-Lifshitz-Gilbert (LLG) equations;

- rate equations naturally emerge from the Fokker-Planck equation when the energy barrier of the DWS is significantly larger than thermal energy $k_BT$, a condition easily fulfilled by magnetite nanoparticles of sufficiently large size ($D \gtrapprox$ 10 nm using the above reported values of magnetic parameters);

- the driving-field frequency *f* is obviously limited by the attempt frequency of the Arrhenius expression for energy barrier crossing, $\nu _0 = \tau _0^{-1} \approx 1 \times 10^9$ Hz (Equation 3). In fact, a frequency significantly lower than $\nu _0$ (i.e., $f \lessapprox 2 \times 10^8$ Hz) should be used, in order to fulfil the condition of detailed balancing^22^.

### Ideal square-wave field: properties and effects

Typically, the high-frequency magnetic fields applied to magnetic nanoparticles in biomedical applications such as magnetic hyperthermia^38,39^ or in imaging/spectroscopy applications (MPI/MPS)^10,12^ have sinusoidal waveform. Although such a choice has technical advantages (e.g., there exist a variety of ways to generate a sinusoidal field of desired amplitude and frequency^40^), it was theoretically demonstrated^22^ and experimentally verified^23–25^ that a non-sinusoidal waveform of *H*(*t*) may produce hysteresis loops characterized by a significantly larger area and a quicker inversion of the magnetization, with beneficial effects on the performance of magnetic particles when they act both as point-like sources of heat in a tissue^38,39^ and as sensing elements^10^.

**Figure 1:**
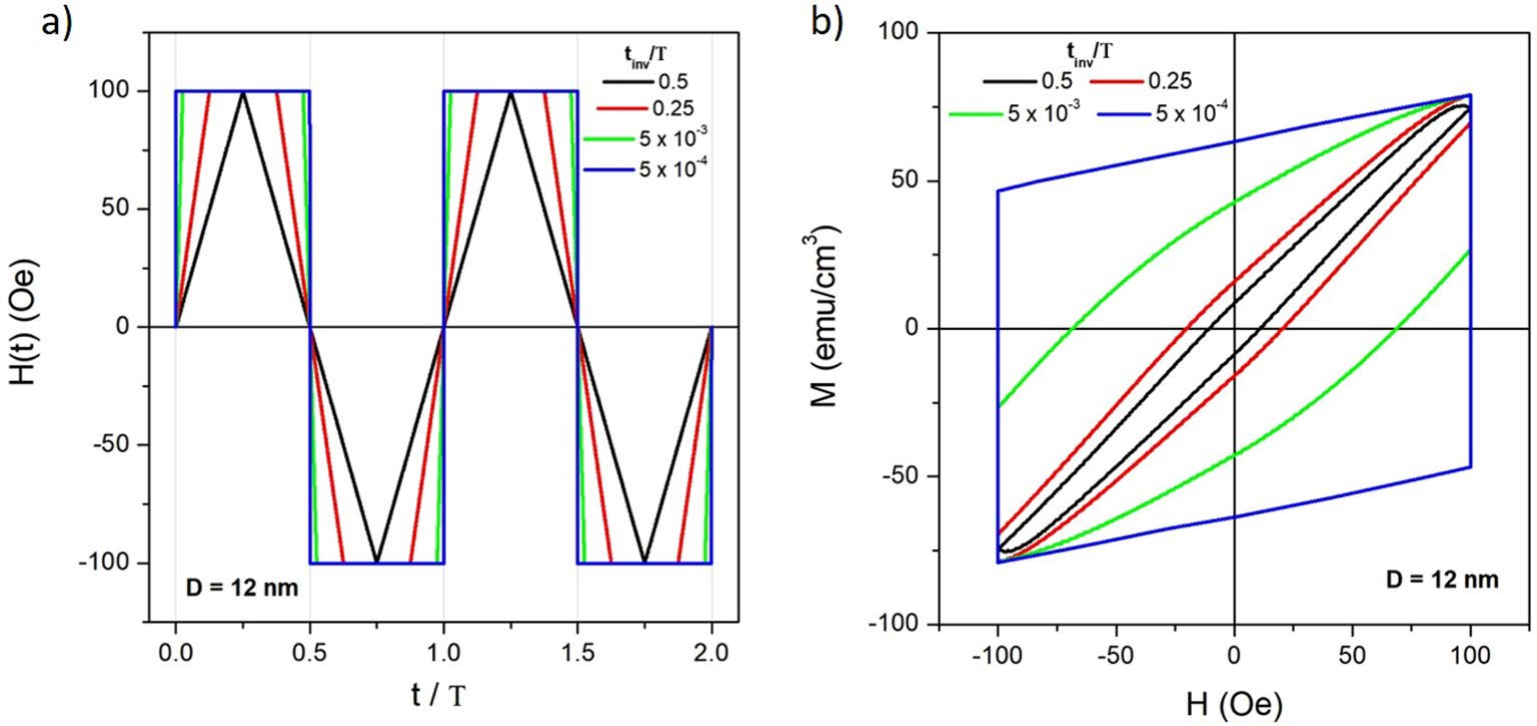
(**a**) Non-conventional magnetic field waveforms characterized by different values of the inversion time $t_{inv}$ with respect to the field’s period *T*; (**b**) minor hysteresis loops corresponding to the four waveforms shown in (**a**) at $f=100$ kHz for 12-nm magnetite nanoparticles whose easy axis makes an angle $\phi =\pi /3$ with the field.

This effect can be clearly observed in Fig. 1, where four ideal waveforms of the magnetic field (triangular, trapezoidal, and square-wave) having same frequency and amplitude are shown, together with the resulting hysteresis loops obtained by numerically solving the rate equations for magnetite nanoparticles at room temperature (panels *a* and *b*, respectively). In this case, $\phi =\pi /3$ and $f=100$ kHz.

The four waveforms correspond to different values of the ratio between the inversion time of the field ($t_{inv}$) and the period $T=1/f$. Such a ratio takes values between 0 and 1/2; the triangular waveform (whose effects are similar to the ones of a sinusoidal waveform) corresponds to $t_{inv}/T=1/2$, whilst the two trapezoidal waveforms are characterized by lower values of this ratio. The ideal SW field can be viewed as the limiting case of an inversion time $t_{inv}$ which becomes vanishingly small with respect to the field’s period.

The resulting hysteresis loops (panel *b*) obtained for magnetite particles of diameter $D=12$ nm display an increasing width and an increasing length of the vertical sides with decreasing the $t_{inv}/T$ ratio. For trapezoidal/SW fields, the vertical sides of the loop appear at $H = \pm H_V$: there, the magnetization is continuously relaxing towards equilibrium with time constant $\tau$ while the magnetic field stays constant for a time depending on the $t_{inv}/T$ ratio. In the SW limit, the hysteresis loop takes the simple form of a regular parallelogram whose vertical sides turn out to be connected by two inclined straight lines corresponding to the abrupt change of the magnetization by effect of the inversion of *H*(*t*).

### Solutions of the rate equation

The features of the SW magnetic field allow us to derive simple analytical solutions for the evolving magnetization. Particles having an easy axis which makes an angle $\phi$ with the field’s direction will be treated before extending the model to the general case of random easy-axis directions.

#### Particles with collinear easy axes

When the nanoparticles have collinear easy axes making the same angle $\phi$ with the field direction ($0 \le \phi \le \pi /2)$, the rate equation for $n_1(t)$ (Equation 1) admits a simple analytical solution under an ideal SW field ($t_{inv} \rightarrow$ 0). After each instantaneous inversion of the field the populations in the two wells relax towards equilibrium starting from initially off-equilibrium values. During each half-period of the square wave, the field has constant amplitude; as a consequence, Equation 1 reduces to a first-order differential equation with constant coefficients, so that the solution $n_1(t)$ follows an exponential law. Using the relation between the value of $n_1$ at equilibrium and the time constants $\tau _i$, which is obtained by setting $dn_1/dt=0$ in the rate equation, i.e.:

8 $$\begin{aligned} n_{1eq}=\tau /\tau _2=\tau _1/(\tau _1+\tau _2) \end{aligned}$$

the evolution of $n_1$ with time during a half period of the square wave (where the field can be either positive or negative) is:

9 $$\begin{aligned} n_1(t')= n_{10}e^{-t'/\tau }+n_{1eq}(1-e^{-t'/\tau }) \hspace{35pt} (0< t' < T/2) \end{aligned}$$

where $t'$ is the time elapsed since the field inversion and $n_{10}$ is the value of $n_1$ when the field inversion occurs; Equation 9 indicates that $n_1(t)$ begins to relax towards the equilibrium value corresponding to the new value of the field. The equation holds as long as *H* stays constant ($= + H_V$ or $- H_V$). It should be noted that at the end of the half-period the equilibrium value $n_{1eq}$ can be either reached or not, depending on the value of the ratio $T/\tau$, i.e., on the rapidity of the particles to redistribute between the two wells according to the new conditions. In particular, the equilibrium value is expected to be attained when $\tau<<T$.

**Figure 2:**
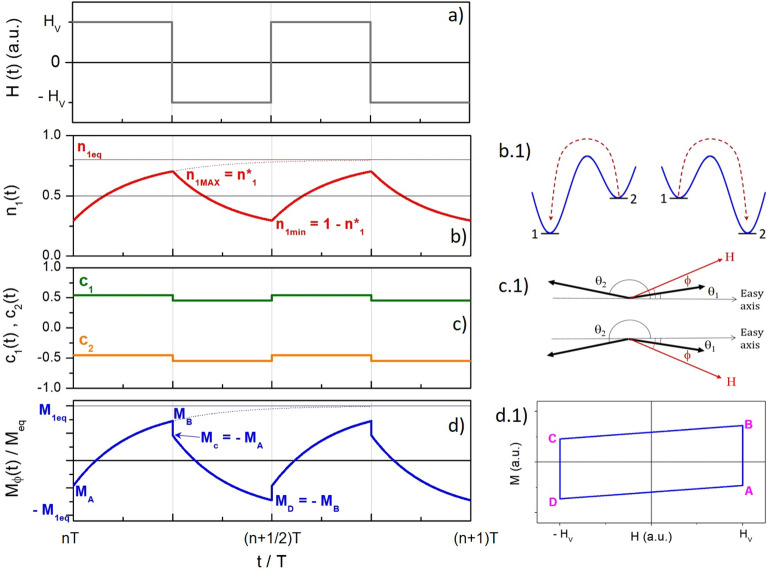
(**a**) time behaviour of the SW field; (**b**) resulting relaxation of the reduced occupancy number $n_1$ by effect of the activated processes sketched in panel (**b.1**); (**c**) changes of the parameters $c_1$, $c_2$ by effect of the rotation of the angles of tilt, as sketched in (**c.1**); (**d**) time behaviour of the magnetization; (**d.1**) resulting minor hysteresis loop. Magnetite nanoparticles, $\phi =\pi /3$.

Figure 2 provides a representation of the behaviour of the SW magnetic field (panel *a*) and of the relaxing occupancy number $n_1(t)$ (panel *b*), studied over two periods between times $t=nT$ and $t=(n+2)T$ (*n* being a positive integer). The initial time is when the square-wave magnetic field *H*(*t*) is turned on; it is supposed that the number of repetitions *n* is so large that the steady state condition has been reached.

The reduced occupancy number $n_1(t)$ changes by effect of the change in shape of the energy profile of the DWS resulting from the periodic inversion of the field, as sketched in panel *b.1*. In the present example, the ratio $T/\tau$ is such that the equilibrium value $n_{1eq}$ under a positive field (red dotted line in panel *b*) is not attained after a half period. In general, when the time *t* is equal to an integer number of half periods, $n_1$ takes maximum (minimum) values equal to $n_{1 MAX} = n_1^* \, (\le n_{1eq})$ and $n_{1 min} = 1-n_1^*$ .

The $n_1(t)$ function shown in Fig. 2b is the steady-state solution of the rate equation, which applies when the square wave field is acting on the DWS since a time much longer that the period *T*. The transient effect occurring just after the square-wave field has been switched on is studied in the Appendix B provided in the Supplementary Information; the calculation reported there allows one to obtain an explicit expression for the quantity $n_1^*$ in steady-state conditions:

10 $$\begin{aligned} n_1^* = \frac{1}{2} \left[ 1-\tanh (T/4\tau ) \right] +n_{1eq} \tanh (T/4\tau ) \end{aligned}$$

The quantity $n_1^*$ is the maximum value of the occupancy number after each half-period of positive field; it is easy to show that when the field is negative the same quantity takes the value $1-n_1^*$. It should be noted that $n_1^* \rightarrow 1/2$ when $\tau>> T$ because in this case the hyperbolic tangent goes to zero, and $n_1^* \rightarrow n_{eq}$ when $\tau<< T$ and $\tanh (T/4\tau ) \rightarrow 1$.

As previously commented, after each inversion of the field the magnetization is affected not only by the relaxation of $n_1(t)$ but also by the sudden rotation of the angles of tilt $\theta _i$, involving a change of the angular parameters $c_1,c_2$ appearing in the expression of $M_{\phi }(t)$ (see Equation 7). This effect is shown in Fig. 2c for $\phi =\pi /3$ and sketched in panel *c.1*. Here, the change of the $\theta _i$ angles is assumed to occur in a vanishingly small time (see below the subsection on “fast” and “slow” effects in the magnetization variation for an in-depth analysis of this assumption).

As a consequence, the evolving magnetization defined by Equation 7 is given by the superposition of a square-wave signal associated to the switch of the angles of tilt $\theta _i$ and of an exponential relaxation associated to the redistribution of particles in the two wells, as shown in Fig. 2d. Here, the characteristic values of the periodic $M_{\phi }(t)$ function are labeled as $M_A,M_B,M_C,M_D$: the values $M_B,M_D$ correspond to the maximum/minimum magnetization values and have opposite sign and same magnitude (less than or equal to the equilibrium magnetization ${M}_{eq}=M_s [n_{1eq} \, (c_1-c_2)+c_2 ]$); $M_A,M_C$ correspond to the values taken by the magnetization immediately after the almost instantaneous rotation of the $\theta _i$ angles and have opposite sign and same magnitude as well. In particular, the peak amplitude of the magnetization signal $M_B$ is given by

11 $$\begin{aligned} M_B= M_s \left[ n_1^* (c_1-c_2)+c_2 \right] . \end{aligned}$$

When the magnetization is reported as a function of the SW field as in panel *d.1*, the hysteresis loop is the parallelogram $\overline{ABCD}$ in the *H*, *M* plane, the vertical segments $\overline{AB}$ and $\overline{CD}$ corresponding to the relaxation of the magnetization at constant field and the inclined straight segments corresponding to the almost instantaneous change of $M_{\phi }$ by effect of the rotation of the angles $\theta _i$. The validity of the present scheme is supported by the perfect agreement between the theoretically predicted shape of the hysteresis loop and the result obtained by numerically solving the rate equations, as shown in the Supplementary Information.

When the easy axes of particles are all parallel to the magnetic field ($\phi =0$), as it is sometimes realized in practice^41–44^, Equation 7 takes a particularly simple form, $c_1$ and $c_2$ being always constant in this case (in fact, $\theta _1=0, \theta _2=\pi$), so that the time behaviour of the magnetization is fully determined by the one of $n_1(t)$ without the effects related to the rotation of the angles of tilt $\theta _i$.

#### Particles with random easy-axis directions

In the more realistic case of independent magnetic nanoparticles with random easy-axis directions in three dimensions, the expressions obtained in the previous section need to be averaged over all directions in space using spherical polar coordinates.

The cylindrical symmetry of the problem makes it possible to average the magnetization $M_{\phi }(t)$ (Equation 7) over the polar angle $\phi$ only:

12 $$\begin{aligned} M(t)=\frac{1}{2}\int _0^{\pi }M_{\phi }(t) \sin \phi \, d\phi \end{aligned}$$.

**Figure 3:**
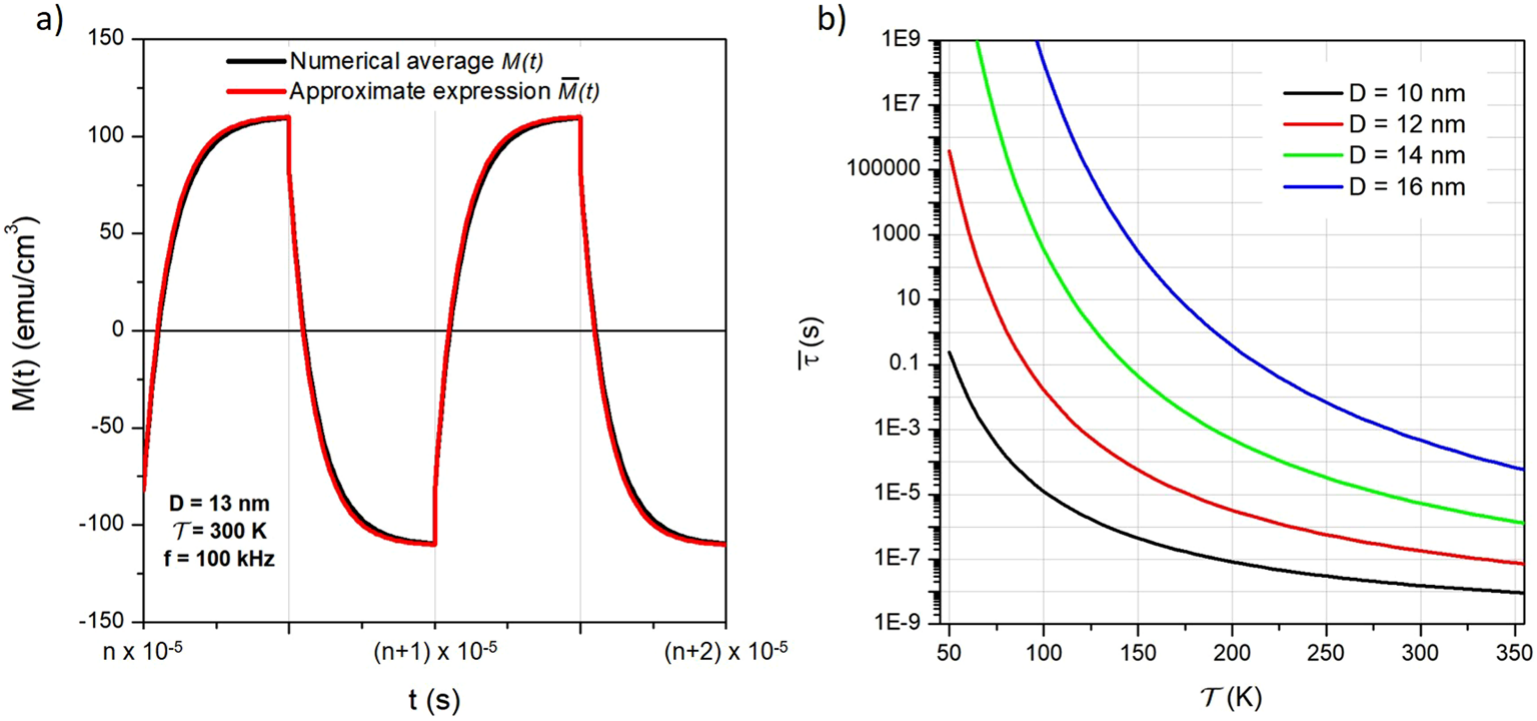
(**a**) Average over all $\phi$ angles of the time behaviour of magnetization; black line: result of numerical calculation of Equation 12; red line: approximate expression (Equation 13); **b**): average relaxation time $\overline{\tau }$ as a function of temperature for selected diameters of the magnetite nanoparticles.

As an example, the *M*(*t*) function resulting from the numerical average of $M_\phi (t)$ over the $\phi$ angles is shown in Fig. 3a for magnetite particles with diameter $D=13$ nm submitted to a SW magnetic field of amplitude $H_V=100$ Oe and frequency $f=100$ kHz (black line). Strictly speaking, while the relaxation of $M{_\phi }$ follows an exponential law for all $\phi$ angles, the average is no longer an exponential curve. An example of the behaviour of $M_{\phi } (t)$ for selected values of the angle $\phi$ and of the average curve *M*(*t*) is given in the Supplementary Information.

However, the *M*(*t*) function defined in Equation 12 turns out to be very well approximated by the following expression:

13 $$\begin{aligned} \overline{M}(t)=M_s\left[ \overline{n}_1(\overline{c}_1-\overline{c}_2)+\overline{c}_2 \right] \end{aligned}$$

which keeps the same form as Equation 7 and where all $\phi$-dependent terms are substituted by their angular averages:

14 $$\begin{aligned} \overline{g} = \frac{1}{2}\int _0^{\pi }g_{\phi } \, \sin \phi \, d\phi \end{aligned}$$

$g_{\phi }$ being any of the $\phi$-dependent quantities $n_1, n_{1eq}, \tau , c_1,c_2$. All $\phi$-averaged quantities to the right side of Equation 13 are functions of time. The $\overline{M}(t)$ function for magnetite nanoparticles with $D=13$ nm is reported in Fig. 3a (red curve): the agreement with the exact *M*(*t*) curve is excellent (and is such for all particle diameters), allowing us to confidently use everywhere the approximate expression in place of the exact one. As a consequence, all the expressions developed for particles with collinear easy axes can be exploited; in particular, the relaxation of $\overline{M}(t)$ related to the evolution of the average occupancy number $\overline{n}_1(t)$ can still be considered to follow an exponential law, and the peak magnetization is given by:

15 $$\begin{aligned} \overline{M_B}=M_s \left[ \overline{n}_1^* \, (\overline{c}_1-\overline{c}_2)+\overline{c}_2 \right] . \end{aligned}$$

where:

16 $$\begin{aligned} \overline{n}_1^* = \frac{1}{2} \left[ 1-\tanh (T/4\overline{\tau }) \right] +\overline{n}_{1eq} \tanh (T/4\overline{\tau }). \end{aligned}$$

Therefore, $\overline{M}_B$ can be expressed as:

17 $$\begin{aligned} \overline{M}_B=\frac{M_s}{2} \, (\overline{c}_1+\overline{c}_2)\, \left[ 1-\tanh (T/4\overline{\tau } ) \right] + \overline{M}_{eq} \tanh (T/4\overline{\tau }) \end{aligned}$$

where $\overline{M}_{eq}=M_s \Big [\overline{n}_{1eq} \, (\overline{c}_1-\overline{c}_2)+\overline{c}_2 \Big ]$. An expression for $\overline{M}_A$ can be obtained along the same lines; as it turns out:

18 $$\begin{aligned} \overline{M}_A=\frac{M_s}{2} \, (\overline{c}_1+\overline{c}_2)\, \left[ 1+\tanh (T/4\overline{\tau } ) \right] - \overline{M}_{eq} \tanh (T/4\overline{\tau }) \end{aligned}$$

Therefore, the length of the vertical sides of the hysteresis loop, $\Delta \overline{M} = \overline{M_B}-\overline{M_A}$ (see Fig. 2d.1) is:

19 $$\begin{aligned} \Delta \overline{M} = \left[ 2\overline{M}_{eq} - M_s \, (\overline{c}_1+\overline{c}_2) \right] \tanh (T/4\overline{\tau }) \end{aligned}$$

The quantity $\Delta \overline{M}$ can be shown to be positive or at least equal to zero.

Finally, the time evolution of the magnetization during the first half-period following the inversion of *H*(*t*) from $-H_V$ to $+H_V$ can be explicitly written as:

20 $$\begin{aligned} \overline{M}^{(A \rightarrow B)}(t')=-\frac{[2\overline{M}_{eq}-\overline{M}_s(\overline{c}_1+\overline{c}_2)]}{1+e^{-T/2\overline{\tau }}} \, e^{-t'/\overline{\tau }}+\overline{M}_{eq} \end{aligned}$$

where $t'$ is the time elapsed from the inversion ($0< t' < T/2$) and the superscript $(A\rightarrow B)$ indicates that this expression is valid in the first half period corresponding to the relaxation from point *A* to point *B* in the hysteresis loop. In the second half period ($T/2< t' < T$), one simply has (with obvious notations): $\overline{M}^{(C\rightarrow D)}(t')= -\overline{M}^{(A\rightarrow B)}(t'-T/2)$. The $\overline{M}(t)$ function can be checked to take the correct values at the characteristic times $t'=0, T/2, T$.

#### “Fast” and “slow” effects in the magnetization variation

A feature of the present model is the sharp separation between the two physical processes which concur to modify the nanoparticle magnetization after an inversion of the magnetic field: *a*) the rotation of the magnetic moments towards the new angles of tilt $\theta _i$ and *b*) the redistribution of the populations of the two energy wells through activated barrier crossing. Such a view makes sense only if the rotation of the magnetization can be safely assumed to be almost instantaneous with respect to the activated relaxation of $\overline{n}_1$.

As a matter of fact, the rotation of the magnetic moment in a nanostructure is expected to be a fast process. Such a concept is supported by studies of the fast or ultra-fast magnetization dynamics in various nanostructures, including magnetic nanoparticles. The rotation, or switch, of the magnetization in a nanostructure by effect of an almost instantaneous external disturbance takes place in a time of the order of a few picoseconds up to about one nanosecond, as demonstrated by the results of theories^45^ or simulations based on the LLG equation of micromagnetics^46–48^ and by a number of advanced measurements^49–51^.

On the other hand, the relaxation of $\overline{n}_1$ takes place in a time determined by the effective time constant $\overline{\tau }$, whose lower limit is in principle the pre-exponential term of the Arrhenius kinetics, $\tau _0 \approx 1$ ns. However, when the rate-equation treatment is applied to magnetic nanoparticles such a theoretical limit cannot be reached by far, as discussed in detail elsewhere^22,36^ on the basis of methods derived from the theory of stochastic processes^52^. The shortest time which can be safely examined by means of the rate equations for magnetic nanoparticles turns out to be of the order of 10 nanoseconds^22^; shorter times cannot be investigated by this method, because the rate equations cease to be a valid approximation to the Fokker-Planck equation^36,52^. Looking at the quantities which determine the effective relaxation time $\overline{\tau }$ (i.e., temperature and energy barrier height) such a requirement rules out particle diameters below 10 nm at room temperature using the present values of the intrinsic magnetic properties of magnetite particles. The variation of the effective time constant $\overline{\tau }$ with temperature is reported in Fig. 3b for different particle diameters.

As a consequence, the separation of the effects of the inversion of the magnetic field between a “fast” effect and a “slow” effect (a rotation of the magnetization vector followed by the relaxation of $\overline{n}_1$) is valid around room temperature for single-core magnetite nanoparticles having diameters $D \ge 10$ nm. This requirement matches the nanoparticles used in most of the current biomedical applications^37^. It is interesting to note that a distinction between “fast” and “slow” magnetization variation at room temperature emerges from the solution of the LLG equation also^47^, i.e., within an independent simulation protocol where the effect of thermal processes is accounted for not in terms of an activated barrier crossing but in terms of an effective thermal field. In that case, the rearrangement of the magnetization by effect of an infinitely fast change of the magnetic field takes place through an adiabatic rotation completed in less than one nanosecond followed by an exponential tail arising from thermal random effects likened to Néel’s relaxation^47^. The agreement between the predictions of the two methods confirms the adequacy of the main assumptions of our model (the macrospin hypothesis and the Néel’s relaxation framework) in the study of magnetic effects occurring in nanoparticles in the considered time range.

Of course, such a sharp separation between “fast” and “slow” effects is not longer possible when a sinusoidal field is applied to the nanoparticles, because under a continuous-wave field the rotations of the magnetization vector and the redistribution of the particles between the two energy wells are intermingled.

## Results

Driving the nanoparticles at high frequency by means of a SW field brings about major advantages which can be acknowledged looking at the main features of the magnetization $\overline{M}(t)$ in both the time and frequency domain. The following results refer to magnetite nanoparticles with random easy-axis directions in three dimensions submitted to a field of magnitude $H_V=100$ Oe. The approximate expression for the average magnetization $\overline{M}(t)$ is used throughout.

### Properties of the solution in the time domain

**Figure 4:**
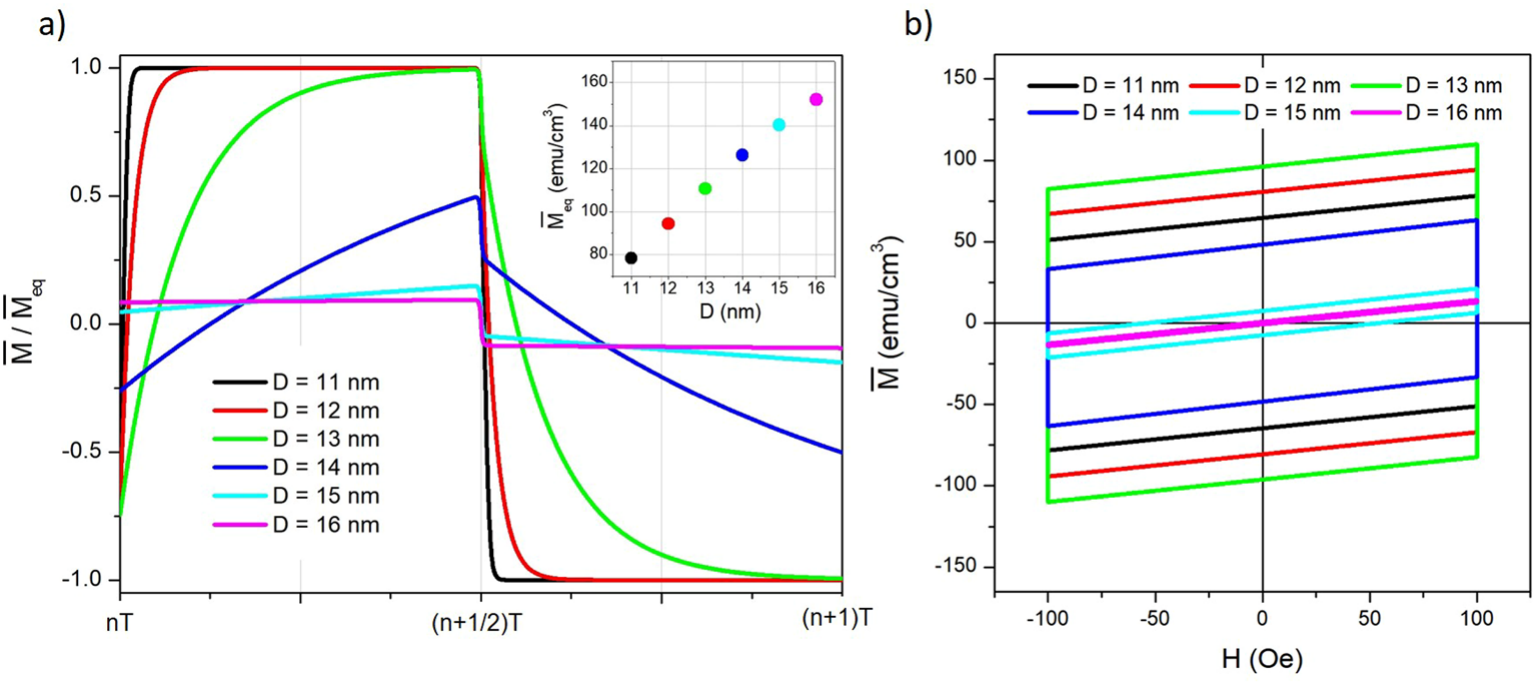
(**a**) Behaviour of $\overline{M}(t)/\overline{M}_{eq}$ over one period for different magnetite nanoparticle diameters; inset: increase of $\overline{M}_{eq}$ with increasing *D*; (**b**) minor hysteresis loops in the ($H,\overline{M}$) plane for the same diameters as in (**a**). Frequency $f=100$ kHz.

The effect of nanoparticle size on the shape of $\overline{M}(t)$ is shown in Fig. 4a, where the steady-state ratio $\overline{M}(t)/\overline{M}_{eq}$ is reported for one full period of the field and for different values of the particle diameter *D*. The field’s period is $T=1/f = 1 \times 10^{-5}$ s and the temperature is $\mathscr {T}=300$ K. The equilibrium value $\overline{M}_{eq}$ is a monotonically increasing function of *D*, as shown in the inset of the same panel.

The absolute value attained by the relaxing magnetization after each half period ($\overline{M}_B$) turns out to be very close to the equilibrium value for $D=11-13$ nm and very distant from it for $D=15$, 16 nm (in this case, it can be observed that $\overline{M}_B$ always stays close to the initial value). This effect depends on the different ratio $(T/\overline{\tau })$, related to the different energy-barrier height which is determined by the particle size, all other parameters being constant in this case.

The corresponding hysteresis loops are shown in panel *b*. For each particle size, the height of the vertical sides of the parallelograms in the (*H*, *M*) plane, $\Delta \overline{M}$, is determined by the greater or less distance of $\overline{M}_B$ from $\overline{M}_{eq}$ and by the magnitude of the equilibrium value itself. A definitely non-monotonic behaviour of the height of the vertical sides with nanoparticle size can be observed in Fig. 4b.

As it is apparent from Fig. 2d.1, the loop’s area $A_L$ is simply equal to the area of a rectangle of height $\Delta \overline{M}$ and length 2$\, H_V$. Using Equation 19 one gets:

21 $$\begin{aligned} A_L= 4 H_V \left[ \overline{M}_{eq} - \frac{M_s}{2} \, (\overline{c}_1+\overline{c}_2) \right] \tanh (T/4\overline{\tau }). \end{aligned}$$

The loop’s area at fixed temperature and frequency displays a maximum at intermediate *D* values, as shown in the Supplementary Information, because for large *D*, $\overline{\tau }>> T$ and $\tanh (T/4\tau ) <<$ 1, while for decreasing *D* the equilibrium magnetization $\overline{M}_{eq}$ steadily decreases as shown in the inset of Fig. 4a.

**Figure 5:**
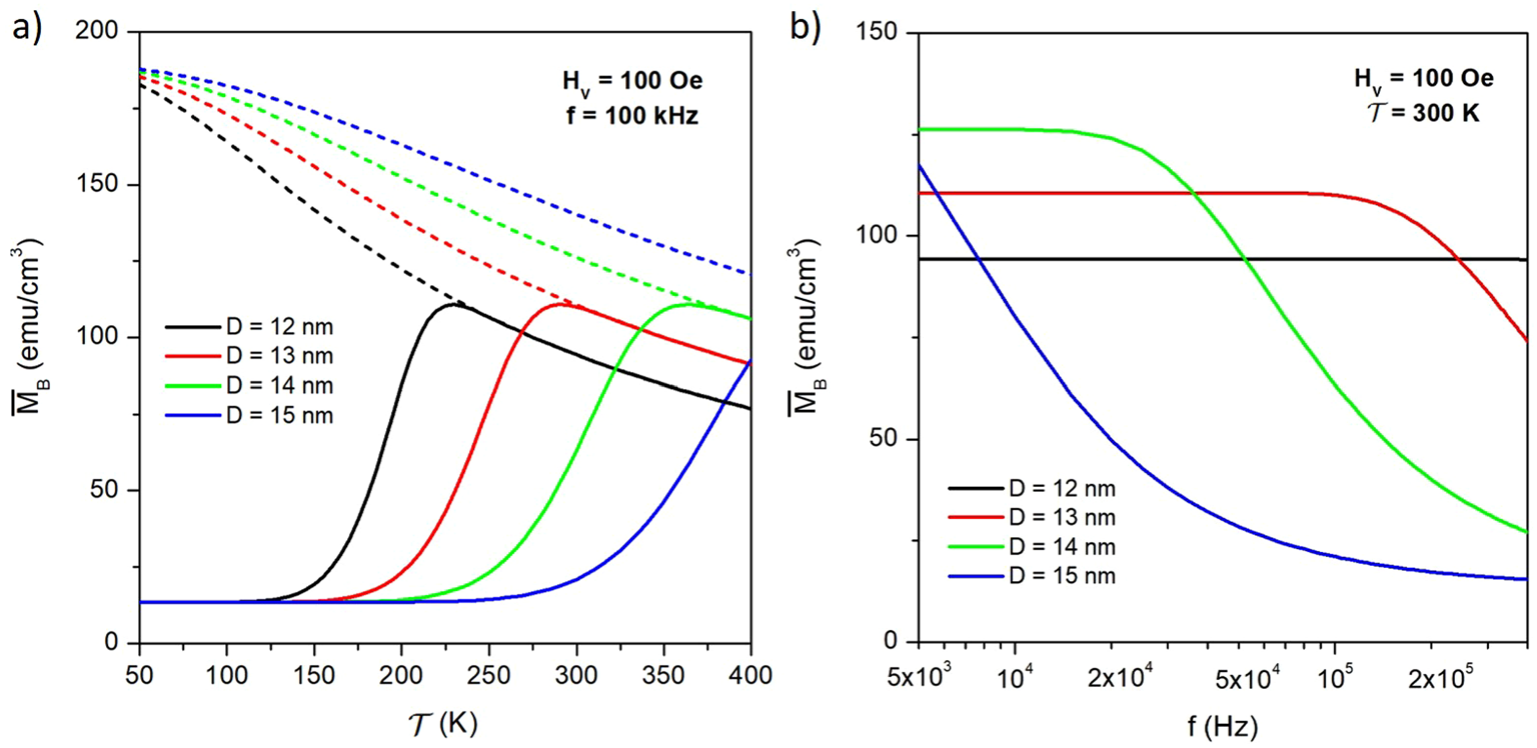
(**a**) peak value of the magnetization signal ($\overline{M}_B$) as a function of absolute temperature $\mathscr {T}$ at $f=100$ kHz for selected nanoparticle diameters (full lines); the corresponding equilibrium curves $\overline{M}_{eq}(\mathscr {T})$ are shown by the dotted lines; (**b**) frequency behaviour of ($\overline{M}_B$) at room temperature for the same nanoparticle diameters as in (**a**).

The peak value of the magnetization signal $\overline{M}_B$ is shown in Fig. 5 for four nanoparticle diameters as a function of absolute temperature $\mathscr {T}$ at $f=100$ kHz (panel *a*) and as a function of frequency *f* at $\mathscr {T}=300$ K (panel *b*). The $\overline{M}_B(\mathscr {T})$ curves start at low temperature from a common, constant baseline equal to $M_s(\overline{c}_1+\overline{c}_2)/2$. because there $\tanh (T/4\tau ) <<$ 1 (see Equation 17); with increasing $\mathscr {T}$, each curve gradually departs from the baseline, increases up to a broad maximum and finally merges with the equilibrium curve $\overline{M}_{eq} (\mathscr {T})$ (dotted lines); this happens when $\overline{\tau }$ becomes so small that the $\tanh (T/4\tau ) \rightarrow$ 1. Such a behaviour takes place in different temperature regions in dependence of the particle diameter. This effect simply reflects the unfreezing of the magnetization of nanoparticles from the blocked to the superparamagnetic (i.e., equilibrium) state, and could be used to simply evaluate the actual blocking temperature of nanoparticles at the operating frequency. The effect of the SW field frequency on the $\overline{M}_B(\mathscr {T})$ curve is shown in the Supplementary Information. Using a very low frequency ($f\approx$ 0.01 Hz), the behaviour of $\overline{M}_B(\mathscr {T})$ allows one to determine the temperature of onset of the superparamagnetic regime of nanoparticles in quasi-static conditions. These results were obtained neglecting as a first approximation the temperature dependence of the intrinsic magnetic properties of nanoparticles.

In the interval *f* = 5–500 kHz, the peak value of the magnetization signal $\overline{M}_B$ displays a sort of complementary behaviour by effect of the continuous variation of the product $(f\tau )=(T/\tau )$, starting from the equilibrium value $\overline{M}_{eq}$ at very low frequencies and decreasing towards a common plateau equal to $M_s(\overline{c}_1+\overline{c}_2)/2$ for $f \rightarrow \infty$. Note that for small particle diameters ($D \le$ 13 nm) the peak magnetization is basically insensitive to the applied frequency over a wide frequency interval.

**Figure 6:**
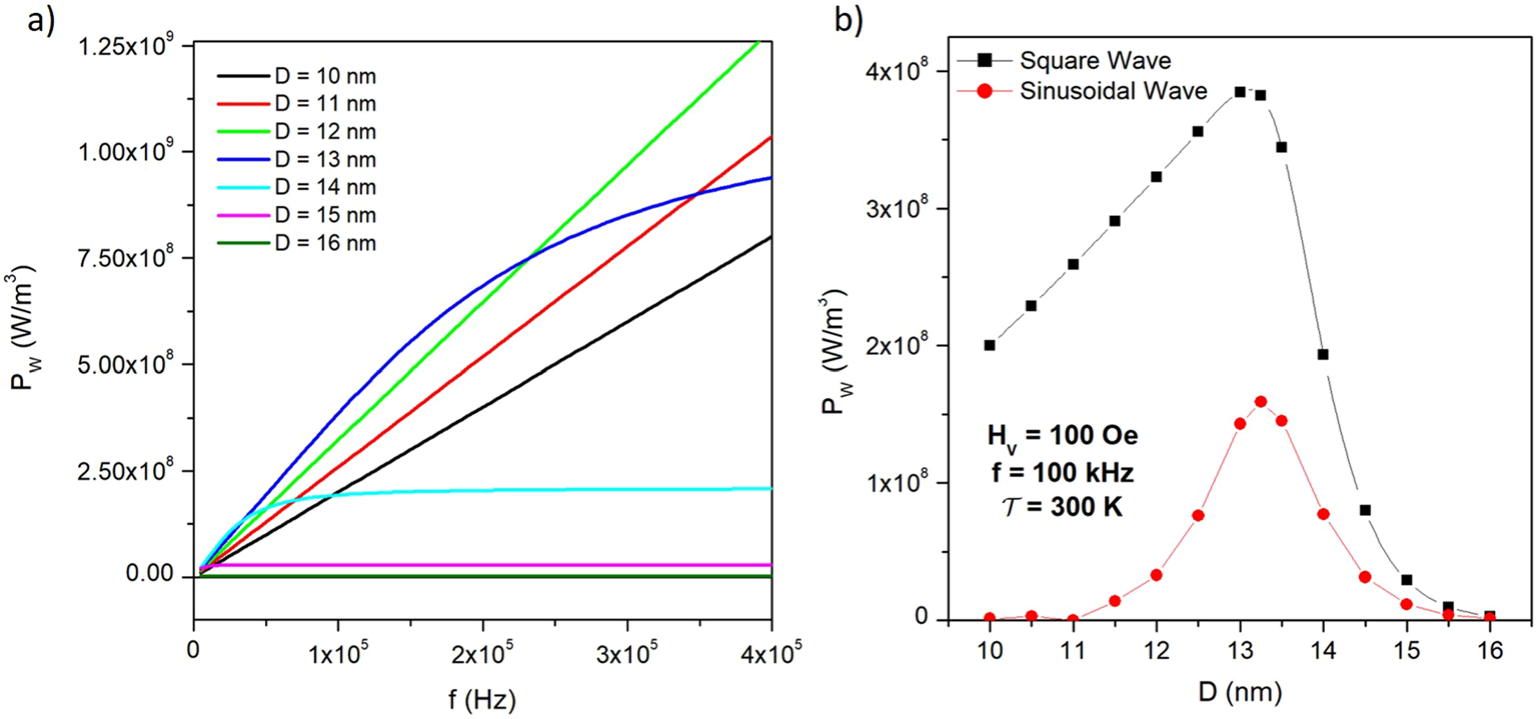
(**a**) behaviour of the magnetic power $P_W$ with frequency for different diameters of the magnetite particles at room temperature; (**b**) magnetic power at $f=100$ kHz for particles of different size submitted to a SW field (black line and symbols) and to a sinusoidal field of same amplitude and frequency (red line and symbols).

The particular features of the loop’s area $A_L$ directly affect the magnetic power $P_W = A_Lf$, i.e., the energy released per unit time by nanoparticles driven at the frequency *f* and exploited to locally heat the environment. It should be recalled that when the concentration of nanoparticles in a tissue is *c* $(0 \le c \le 1)$, the heating power is simply written as $P_W c$; in typical hyperthermia treatments *c* often takes values in the 0.001-0.01 range^20,53–55^. The heating power (in SI units) of magnetite nanoparticles of different diameters is shown in Fig. 6a as a function of the applied frequency in the interval $0 \le f \le$ 400 kHz. For small particles ($D < 13$ nm) the loop’s area is basically constant with frequency because $\overline{M}_B$ is constant (Fig. 5b), so that the heating power is directly proportional to *f* with a slope which increases with *D*. For larger diameters, all $P_W(f)$ curves show a curvature in the considered frequency region; in particular, for $D \ge$ 14 nm an asymptotic plateau of $P_W$ is soon reached, whose magnitude is dramatically reduced at large *D*. This effect is explained considering that for large particles the hyperbolic tangent in the expression for $A_L$ (Equation 21) decreases with increasing frequency as $\tanh (T/4\overline{\tau }) \sim (T/4\overline{\tau }) \sim 1/f$.

The linear behaviour of $P_W$ with *f* in small particles can have significant impact in hyperthermia applications where a suitable choice of the power released by the magnetic particles is of crucial importance for the achievement of the correct temperature of the target tissue: in fact, a simple linear relation between $P_W$ and *f* could be particularly useful to this aim.

The power released by magnetite nanoparticles of different diameters submitted to a SW field at 100 kHz (black line and symbols) is compared in Fig. 6b to the one produced by the nanoparticles under a sinusoidal field of same amplitude and frequency (red line and symbols). For both waveforms the maximum of $P_W$ occurs around the same *D* value; however, the advantage of using the square-wave field is apparent for all diameters. In both cases, $P_W$ disappears above a critical nanoparticle size ($D\approx$ 16 nm for the $K_{eff}$ value considered in this paper) because both energy barriers $E_{B1}$, $E_{B2}$ of the DWS become too large to allow for energy-barrier crossing in one period of the driving field. As a matter of fact, the magnetization of a small particle driven by a continuously evolving field, such a sinusoidal wave, is always close to equilibrium and is therefore very similar to a Langevin curve^56^ which has no hysteresis; on the contrary, when a square-wave field is applied, the instantaneous inversion of *H*(*t*) at every half period produces a wide hysteresis loop, despite the particles are almost at equilibrium.

Finally, it should be noted that the voltage induced by a SW field basically occurs in bursts with an interval of one half-period between subsequent bursts. Detrimental effects such as healthy tissue heating, possibly arising from the eddy currents generated by the induced-voltage bursts, were discussed in a previous paper^22^, where it was shown that using realistic SW waveforms still complies with the requirement of not damaging living bodies or bring discomfort to patients. In fact, the time interval between subsequent induced-voltage bursts - where the magnetic field is kept constant - is long enough to dampen the undesired heating effect of short eddy-current pulses in a self-regulating biological environment.

### Properties of the frequency spectrum

The average magnetization $\overline{M}(t)$ is a periodic function of time where the “fast” effect is depicted as a square wave and the “slow” relaxing effect is well approximated by an exponential curve. Therefore, the Fourier coefficients can be easily calculated, as reported in the Appendix C provided in the Supplementary Information. This is one of the very few cases where it is possible to give analytic formulas for the Fourier coefficients of the magnetization of magnetic nanoparticles, another case being the frequency spectrum of the fully anhysteretic response of ideal Langevin particles^57^.

**Figure 7:**
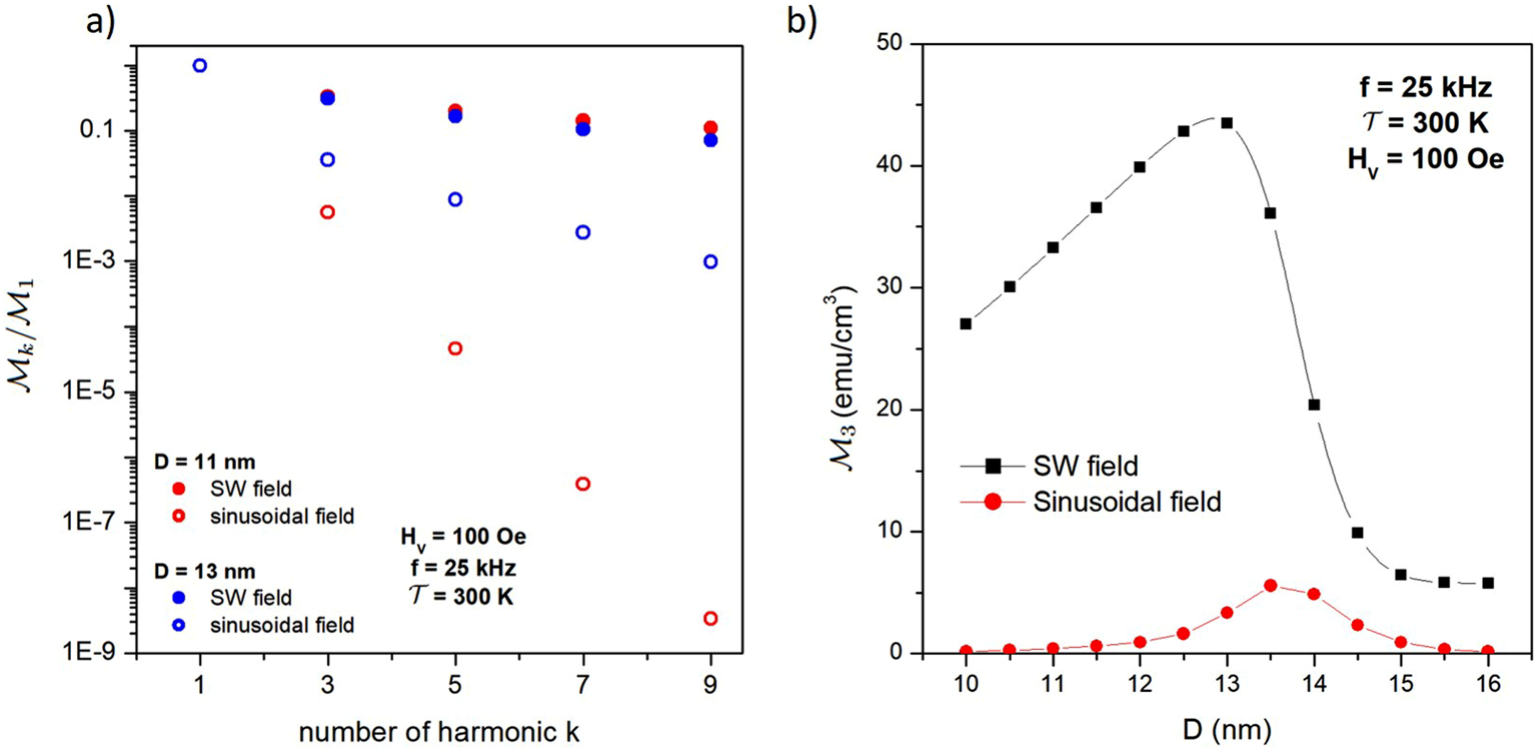
(**a**) magnitude of the first five harmonics of the frequency spectrum $\mathscr {M}_k$ normalized to the magnitude of the first harmonic for two nanoparticles submitted to a SW field (full symbols) and to a sinusoidal field (open symbols); (**b**) magnitude of the third harmonic $\mathscr {M}_3$ for magnetite nanoparticles of different size submitted to a SW field (black symbols and lines) and to a sinusoidal field (red symbols and lines).

The magnitude of the first odd harmonics of the frequency spectrum of magnetization is reported for two nanoparticle diameters in Fig. 7a as the ratio to the magnitude of the first harmonic $\mathscr {M}_k/\mathscr {M}_1$ (full symbols). Here, *k* is an odd integer ($k = (2n+1)$ with $n=0,1,2\ldots$). The frequency of the SW field is $f=25$ kHz, a typical value used in applications such as MPI/ MPS^10,12^. The magnitude of the *k*th harmonic is $\mathscr {M}_k=(P_k^2+Q_k^2)^{1/2}$; the analytical expressions of coefficients $P_k$ and $Q_k$ are given in Equation 5 of Appendix C provided in the Supplementary Information.

For both nanoparticle diameters, $\mathscr {M}_k$ slowly decreases with increasing *k*; the reduction factor of the *k*th harmonic with respect to the first one is rather small, $\mathscr {M}_k$ being still larger than a few percent of $\mathscr {M}_1$ when $k=9$. The decrease of $\mathscr {M}_k$ with *k* is only slightly affected by particle size for the *D* values considered in this paper. On the contrary, when the nanoparticles are submitted to a sinusoidal field of same amplitude and frequency, the behaviour of the ratio $\mathscr {M}_k/\mathscr {M}_1$ with the number of the harmonic *k* becomes much steeper, as it can be observed in Fig. 7a (open symbols in colour). In this case, the harmonics were obtained by using a FFT package after numerically solving the rate equations^58^. It should be noted that while the particles with $D=11$ nm still exhibit a pure superparamagnetic (anhysteretic) behaviour when they are submitted to a sinusoidal field at $f=25$ kHz, the ones with $D=13$ nm are characterized by a non-negligible hysteresis loop^56^. This difference is reflected in the different slope of the corresponding $\mathscr {M}_k/\mathscr {M}_1$ curves (open symbols).

In any case, it is apparent that nanoparticles driven by a sinusoidal field have a much stronger reduction factor of the higher-order harmonics with respect to the first one. A similar strong reduction of the magnitude of the higher-order harmonics of the magnetization with respect to the first one was often observed in measurements on magnetite nanoparticles driven by a sinusoidal field^15,41,59,60^.

It is concluded that substituting the sinusoidal field with a square-wave field of same amplitude and frequency would produce a frequency spectrum of the magnetization particularly rich in higher-order harmonics, resulting in a dramatic increase of their magnitude. This applies to the third harmonic $\mathscr {M}_3$ also, which plays a central role in MPI applications^10,11^.

The behaviour with *D* of the quantity $\mathscr {M}_3$ produced by either a square-wave or a sinusoidal driving field of same amplitude and frequency is shown in Fig. 7b. The overall $\mathscr {M}_3(D)$ curve for the square-wave field is similar to the behaviour of the loop’s area $A_L$ with *D* (see the Supplementary Information). The enhancement of $\mathscr {M}_3(D)$ is apparent, particularly in the low diameter limit.

The significant enhancement of $\mathscr {M}_3$ may have important consequences on the performance of magnetic nanoparticles used as tracers in MPI^10,11,56^, because a much higher value of the third harmonic means a substantially higher sensitivity of the technique, allowing the specialists to achieve the same contrast and definition of the image while using a significantly lower concentration of magnetic nanoparticles.

## Conclusion

The application-oriented properties of magnetic nanoparticles driven by a high-frequency magnetic field can be substantially improved by a suitable choice of the field’s waveform. In particular, applying a SW field to single-core magnetite particles results in a significant enhancement of the high-frequency performance of magnetite nanoparticles as a consequence of the unique behaviour of the cyclic magnetization. In this case, the process is shown to consist of a nearly instantaneous rotation of magnetization at each inversion of the field and a slower activated relaxation towards equilibrium at constant field; such a sharp separation between “fast” and “slow” effects is confirmed by experiments and simulations of the fast and ultra-fast dynamics of magnetization in nanostructures.

A rate equation model has been used to evaluate the effect of a SW field on the time behaviour of the magnetization of magnetic nanoparticles with random easy-axis directions in three dimensions. The rate equations have been easily solved and shown to result in analytic expressions for the time behaviour of the magnetization and for the harmonics of the frequency spectrum.

The most significant consequences of exciting the magnetic nanoparticles by a SW field are a large enhancement of the power released by the nanoparticles at frequencies typical of MH applications and a highly enhanced sensitivity of the same particles at frequencies typical of MPI/MPS. In both cases, the performance turns out to be definitely better than the one of the same particles submitted to a sinusoidal field of same amplitude and frequency. Other interesting results include the simple linear dependence of the magnetic power $P_W$ on frequency for small nanoparticles and the possible use of the peak amplitude of the time-resolved magnetization to find the blocking temperature of the nanoparticles at all frequencies.

Although the present model describes the magnetic response of nanoparticles under the action of an *ideal* SW field (i.e., characterized by $t_{inv} \rightarrow 0$), the results described in this paper may serve as a starting point to understand the behaviour of the magnetization of nanoparticles driven by more realistic SW fields, which are characterized by a finite inversion time.

A comment on this point seems to be appropriate. A SW field of practical interest in biomedical applications of magnetic nanoparticles should have an inversion time $t_{inv}$ as short as possible and an adequate amplitude. Although the SW fields realized so far as limiting cases of trapezoidal waveforms have inversion times of the order of the microsecond, this quantity could be significantly reduced by means of suitable technological improvements of both control electronics and slave circuitry.

In order to preserve the separation between the “fast” change of the angles of tilt $\theta _i$ and the “slow” evolution of $\overline{n}_1$ with time, and therefore make use of the results of the present model, the time $t_{inv}$ taken by the field to change sign should be significantly lower than the time constant $\overline{\tau }$ governing the redistribution of particles within the two energy wells of the DWS. It is suggested that such a condition can be considered as fulfilled when $t_{inv} \le 0.1 \, \overline{\tau }$.

Both the highest magnetic power $P_W$ and the highest magnitude of the third harmonic $\mathscr {M}_3$ are predicted to occur for diameters of 13-14 nm in nanoparticles driven by a SW field (with the present choice of the values of the intrinsic magnetic properties of magnetite nanoparticles). Taking into account the behaviour of $\overline{\tau }$ with particle diameter at room temperature (see Fig. 3b), it can be estimated that an inversion time of about 100 ns would be sufficient to include particle diameters of 13-14 nm in the range of validity of the present model. An inversion time of about 100 ns is estimated to be accessible by making a few improvements to the present-day technology, and would allow the users to take advantage of the improved performance of nanoparticles at their best.

In conclusion, the present theoretical treatment may pave the way to the development of more efficient techniques to magnetically drive the nanoparticles for biomedical applications, with the proviso that the encouraging results described in this paper be viewed as a goal which can be approached to a larger or lesser degree in dependence of one’s technical ability to produce a sharp inversion of the field and of the choice of intrinsic magnetic properties and size of nanoparticles in such a way that the condition $t_{inv}/\overline{\tau } \le$ 0.1 be satisfied.

## Supplementary Information

Supplementary Figures.

Supplementary Information 1.

## Data Availability

The datasets used and/or analysed during the current study available from the corresponding author on reasonable request.
