# Supplementary material for: Magnetic nanoparticles in square-wave fields for breakthrough performance in hyperthermia and magnetic particle imaging
Source: Sci Rep. 2024 May 10;14:10704. doi: 10.1038/s41598-024-61580-8 (PMC11636937; doi:10.1038/s41598-024-61580-8)
Supplement: Supplementary file 1 — Supplementary Figures. [file 41598_2024_61580_MOESM1_ESM.pdf]

Magnetic nanoparticles in square-wave fields for breakthrough performance in hyperthermia and magnetic particle imaging

G. Barrera, Paolo Allia, Paola Tiberto

INRIM

Advanced Materials Metrology and Life Sciences Turin, Italy

Supplementary Information

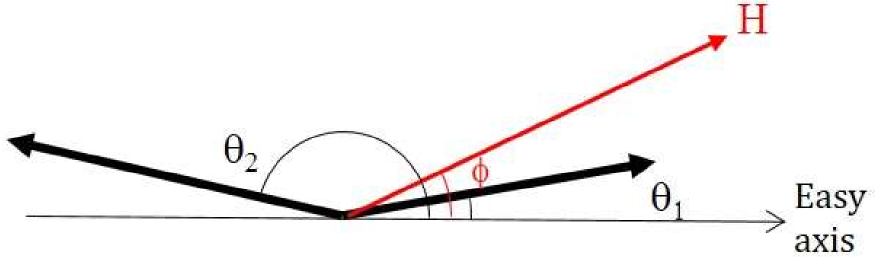

Figure SM1: Sketch of the angles involved in the process of magnetization of a nanoparticle. At zero applied field the magnetization is aligned to the horizontal easy axis (pointing either to the right or to the left); when a magnetic field is applied at an angle ϕ with the easy axis, the magnetization rotates towards the angles of tilt θ_1 and θ_2, which are determined by the condition of minimum magnetic energy.

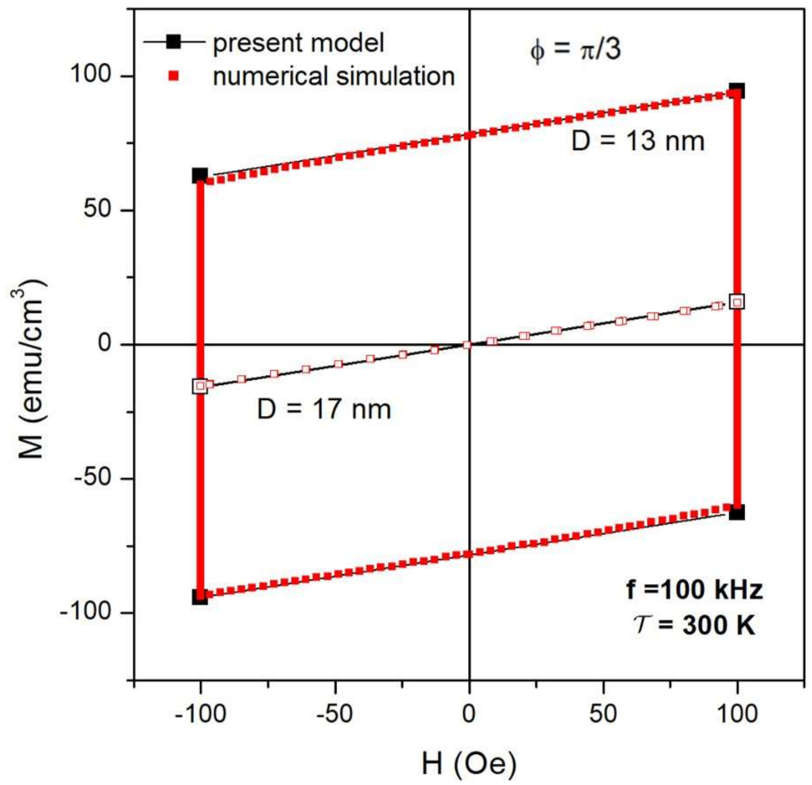

Figure SM2: Comparison between the hysteresis loops predicted by the model for an ideal SW field and the results of a numerical solution of the rate equations for two diameters of magnetite nanoparticles. For the particles with D = 17 nm the hysteresis loop is in practice reduced to a line. The agreement between the model and the simulation is excellent, in spite of the fact that the numerical simulation has been obtained using a finite inversion time (responsible for the small downward bending of the simulation.

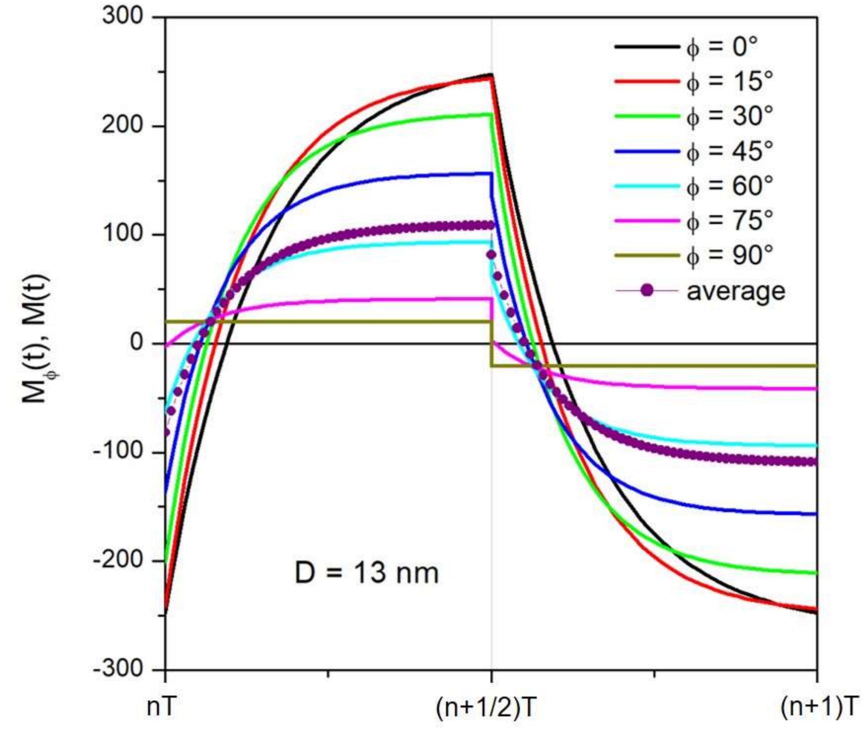

Figure SM3: Behaviour of the room-temperature magnetization $M_\phi(t)$ over one period of the SW field for collinear magnetite nanoparticles whose easy axis makes different angles ϕ with respect to the magnetic field. Note that when ϕ=0°, there is no magnetization jump after one half period (because in this case there is no rotation of the tilt angles θ_i); when ϕ=90° the magnetization is a square wave (because in this case there is no relaxation of n_1). The average of $M_\phi(t)$ over all ϕ angles is also reported (full dots).

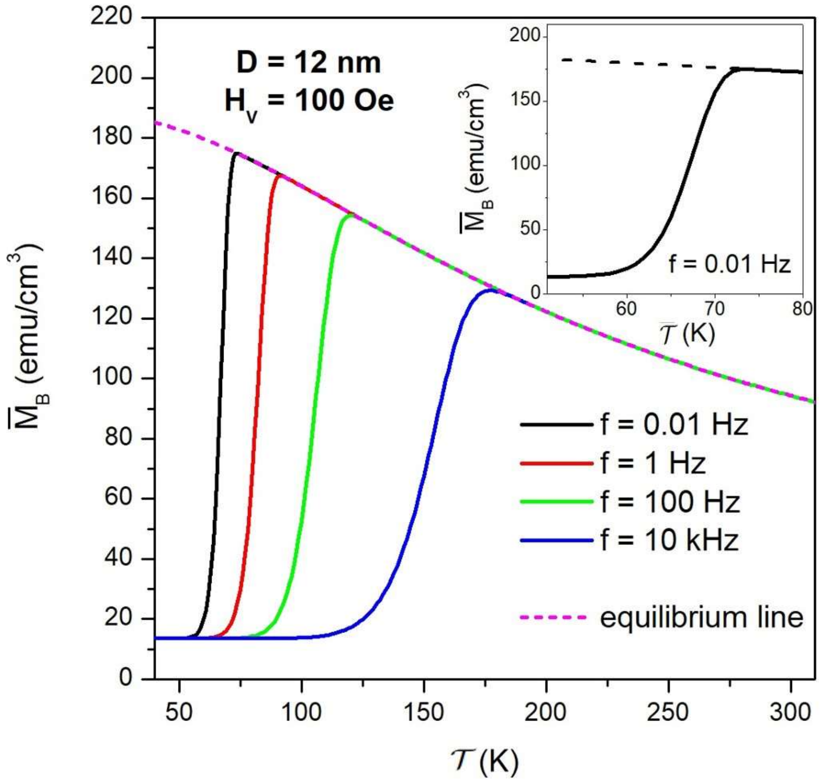

Figure SM4: Temperature behaviour of the peak value of the magnetization signal resolved in time (M_B) for a 12-nm magnetite nanoparticle at different SW field frequencies. The curve for f = 0.01 Hz corresponds to the quasi static case and can be used to precisely define the blocking temperature of nanoparticles; the sharp transition resulting in this case region is magnified in the inset.

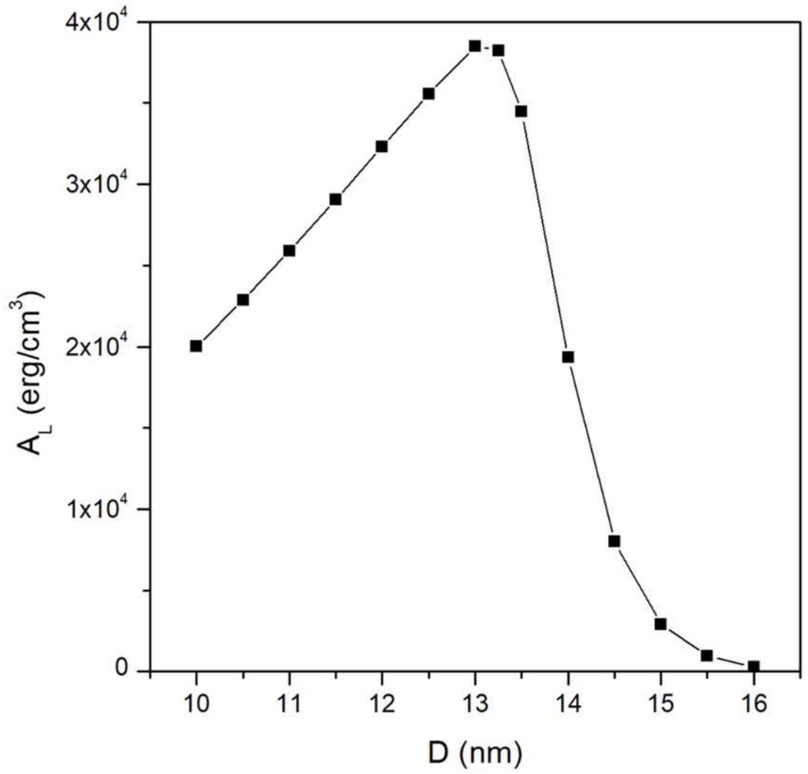

Figure SM5: Loop's area A_L as a function of diameter for magnetite nanoparticles at room temperature. SW field frequency: 100 kHz.

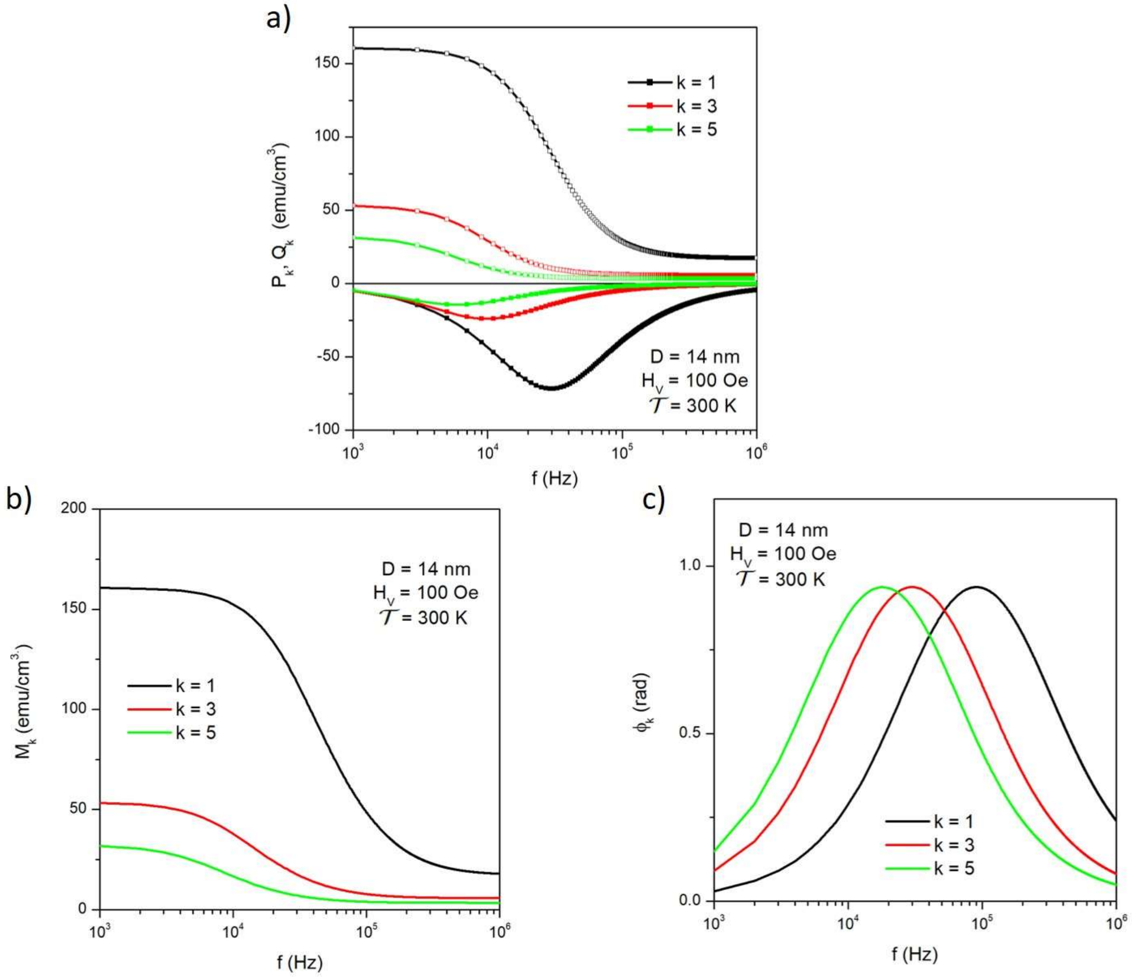

Figure SM6: Frequency behaviour of the first three harmonics of the magnetization spectrum for a 14-nm magnetite nanoparticle: a): P_k, Q_k coefficients; b): magnitude; c) phase.
