# Supplementary material for: Magnetic nanoparticles in square-wave fields for breakthrough performance in hyperthermia and magnetic particle imaging
Source: Sci Rep. 2024 May 10;14:10704. doi: 10.1038/s41598-024-61580-8 (PMC11636937; doi:10.1038/s41598-024-61580-8)
Supplement: Supplementary file 2 — Supplementary Information 1. [file 41598_2024_61580_MOESM2_ESM.pdf]

Magnetic nanoparticles in square-wave fields for breakthrough performance in hyperthermia and magnetic particle imaging

G. Barrera, P. Allia, P. Tiberto

INRiM, Advanced Materials Metrology and Life Sciences, Torino, Italy

Appendix A

The time constants τ_i can be written as

$$\tau_i = \tau_0 \exp\left(\frac{E_{Bi}}{k_B \mathscr{T}}\right) = \tau_0 \exp\left(\frac{E_M - E_i}{k_B \mathscr{T}}\right) \qquad (i = 1, 2) \tag{1}$$

where the energy barrier E_{Bi} is the difference $(E_M - E_i)$ between the energy of the maximum between the wells and the minimum i. It should be noted that all the quantities E_M, E_1, E_2 are dependent on the magnitude of the applied field $|H_V|$. Using Equation 2 of the main text, one easily gets:

$$\tau = \tau_0 \exp\left(\frac{E_M}{k_B \mathscr{T}}\right) \frac{\exp\left[\frac{(E_1+E_2)}{k_B \mathscr{T}}\right]}{\exp\left[\frac{E_1}{k_B \mathscr{T}}\right] + \exp\left[\frac{E_2}{k_B \mathscr{T}}\right]} \equiv \tau_N \frac{\exp\left[\frac{(E_1+E_2)}{k_B \mathscr{T}}\right]}{\exp\left[\frac{E_1}{k_B \mathscr{T}}\right] + \exp\left[\frac{E_2}{k_B \mathscr{T}}\right]} \equiv \tau_N \beta(|H_V|) \qquad (i = 1, 2) \tag{2}$$

The behaviour of the $\beta(|H_V|)$ function is reported in Figure 1 for particles whose easy axis makes an angle $\phi = 60°$ with the magnetic field directions (similar results are found for different ϕ angles). Selected values of the particle diameter are considered.

The $\beta(|H_V|)$ curves always start from the value $\frac{1}{2}$ for $|H_V| = 0$ and monotonically decrease with increasing $|H_V|$; their slopes are increasingly steeper with increasing particle size. It should be noted that the lower is β, the smaller is the effective time constant τ, and the faster is the relaxation of magnetization. Generally speaking, therefore, the presence of a magnetic field always makes the relaxation faster than predicted by the usual expression for the Néel's relaxation, τ_N (which holds when $|H_V| = 0$ only).

The effect of nanoparticle size on β is shown in Figure 2 for three values of the driving field magnitude. The angle ϕ between easy axis and magnetic field is the same as in Figure 1. The parameter β is always a decreasing function of D, i.e., larger particles are characterized by a faster relaxation of the magnetization by effect of the same driving-field magnitude.

Appendix B

In collinear nanoparticles (same ϕ angle) the expression for the peak amplitude of the magnetization signal in steady-state conditions (the value labeled as M_B in panel d of Figure 2 in the main text) can be obtained by analysing the behaviour of the reduced occupancy number $n_1(t)$, as determined by the rate equations for a given ϕ angle, starting from the time $t = 0$ when the square-wave magnetic field $H(t)$ has been switched on. The initial condition chosen for $n_1(t)$ is $n_1(0) = n_2(0) = 1/2$ (i.e., the two wells are considered to be equally populated, as in the ideally demagnetized state). The quantity $n_1(t)$ follows an exponential law in each half-period of the square wave field; here, we are looking at the value taken by n_1 precisely after one, two, ... m half-periods of the field. It is convenient to introduce the auxiliary quantity $x(t) = [n_1(t) - 1/2]$ which takes values between $-1/2$ and $1/2$ and whose initial value is $x(0) = 0$.

It is easy to directly verify that the quantity $x(t)$ obtained by solving the rate equation takes the following values x_m after the first four half periods of the field ($m = 1 - 4$; the field is assumed positive in the first half period):

$$x_1 = x_{eq}(1 - e^{-T/2\tau})$$
$$x_2 = -x_{eq}(1 - 2e^{-T/2\tau} + e^{-2T/2\tau})$$
$$x_3 = x_{eq}(1 - 2e^{-T/2\tau} + 2e^{-2T/2\tau} - e^{-3T/2\tau})$$
$$x_4 = -x_{eq}(1 - 2e^{-T/2\tau} + 2e^{-2T/2\tau} - 2e^{-3T/2\tau} + e^{-4T/2\tau})$$

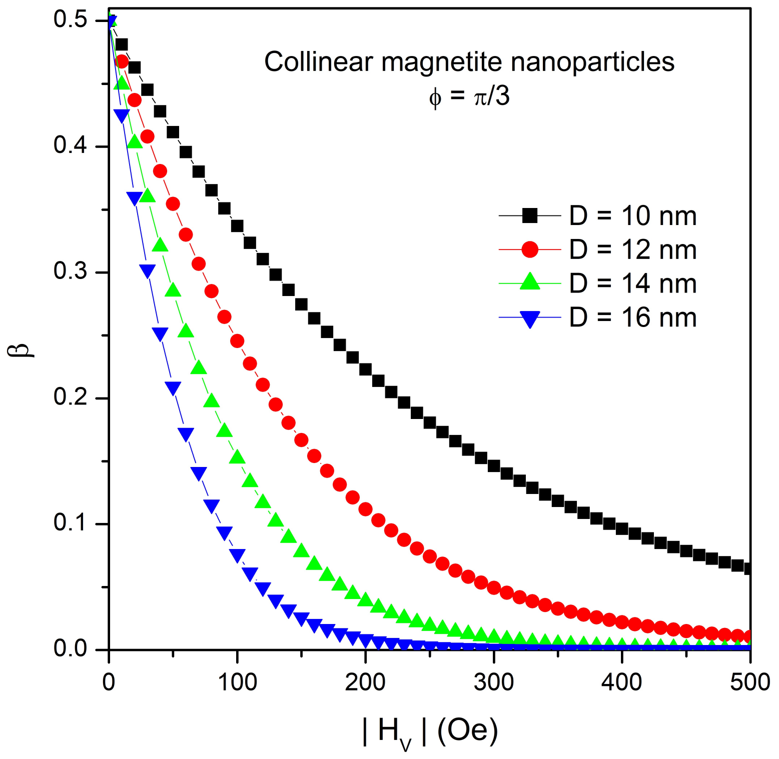

Figure 1. Effect of the magnitude $|H_V|$ of the SW driving field on the function β defined in Equation 2. The $\beta(|H_V|$ curve becomes increasingly steeper with increasing particle size.

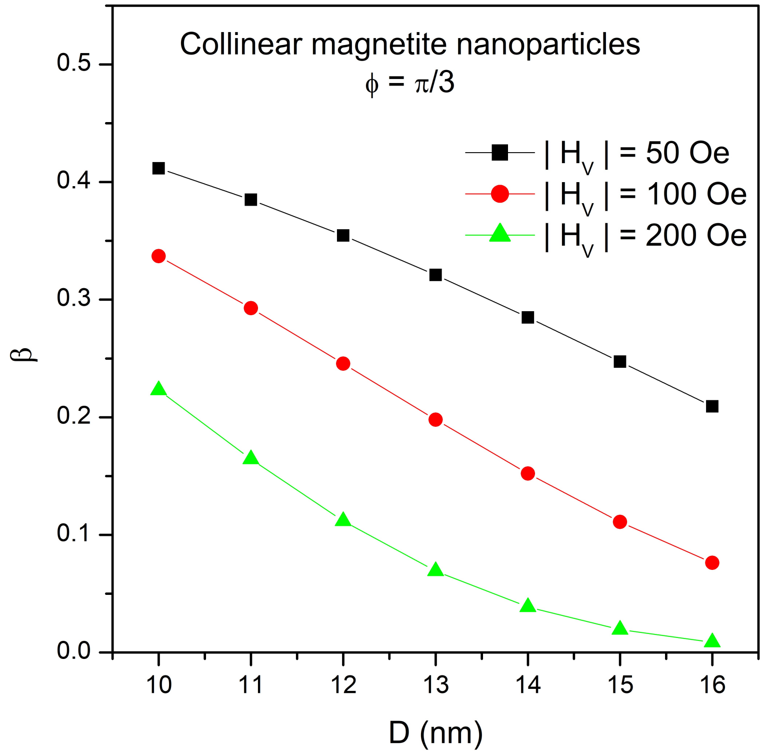

Figure 2. Effect of nanoparticle diameter on the function β defined in Equation 2 for three selected values of the driving field's magnitude $|H_V|$. The β parameter at fixed $|H_V|$ becomes increasingly smaller with increasing particle size.

Here, $x_{eq} = [n_{1eq} - 1/2]$, where n_{1eq} is the equilibrium value of $n_1(t)$ defined by Equation 3 and shown in Figure 2 of the main text, and τ is the effective time constant for the considered ϕ angle. Generally speaking, after $m > 1$ half-periods of the field the quantity x_m takes the following value:

$$x_m = (-1)^{m-1} x_{eq} \Big(1 + 2 \sum_{k=1}^{m-1} (-1)^k e^{-kT/2\tau} + (-1)^m e^{-mT/2\tau} \Big)$$

so that in the limit $m \to \infty$, corresponding to the steady-state condition:

$$x_{m\to\infty} = (-1)^{m-1} x_{eq} \Big[2 \sum_{k=0}^{\infty} (-1)^k e^{-kT/2\tau} - 1 \Big]$$

Note the change in the lower limit of the summation within brackets; such a summation is the geometric series of argument $(-e^{-T/2\tau})$, so that:

$$x_{m\to\infty} = \pm x_{eq} \Big(\frac{2}{1 + e^{-T/2\tau}} - 1 \Big) = \\ = \pm x_{eq} \frac{1 - e^{-T/2\tau}}{1 + e^{-T/2\tau}} = \pm x_{eq} \tanh(T/4\tau).$$

where the $\pm$ sign applies to the half-periods of positive/negative H_V. Recalling that $n_1^* = [x_{m\to\infty} + 1/2]$ and $n_{eq} = [x_{eq} + 1/2]$, one easily obtains the expression for n_1^* given in Equation 5 in the main text and the peak magnetization M_B of Equation 6 in the main text .

In the case of particles with random easy-axis directions, the above results still hold having care of using the average values instead of the ϕ-dependent ones.

The initial transient of the quantity $\overline{n}_1^*$, i.e., the relaxation of $\overline{n}_{1m}$ towards the steady-state value after m field periods, is shown in Figure 3. The duration of the transient is strongly affected by the ratio $T/\overline{\tau}$; considering the driving-field frequencies used in typical biomedical applications of nanoparticles (which are in the 10-250 kHz range), in all practical cases the steady-state condition is soon reached.

This is not the first occurrence of transient effects emerging from the rate-equation treatment of the cyclic magnetization of magnetic nanoparticles: similar effects were predicted to take place in minor hysteresis loops immediately after the onset of a sinusoidal field[1], and were confirmed by a different probabilistic model[2].

Appendix C

In this work, $H(t)$ is an odd function of time ($H(t) = -H(-t)$) characterized by half-wave odd symmetry (see Figures 1 and 2 of the main text), so that it can be developed in a Fourier sine series containing odd harmonics only:

$$H(t) = \frac{4}{\pi} H_V \sum_{n=0}^{\infty} \frac{1}{2n+1} \sin[(2n+1)\omega t]$$

where $\omega = 2\pi f$. The magnetization $\overline{M}(t)$ is characterized by half-wave symmetry and is expressed as a Fourier series containing odd harmonics only:

$$\overline{M}(t) = \sum_{n=0}^{\infty} P_{2n+1} \cos[(2n+1)\omega t] \\ + \sum_{n=0}^{\infty} Q_{2n+1} \sin[(2n+1)\omega t] \tag{3}$$

Calling $x = \omega t$, the Fourier coefficients of $\overline{M}(t)$ are obtained from the following integrals:

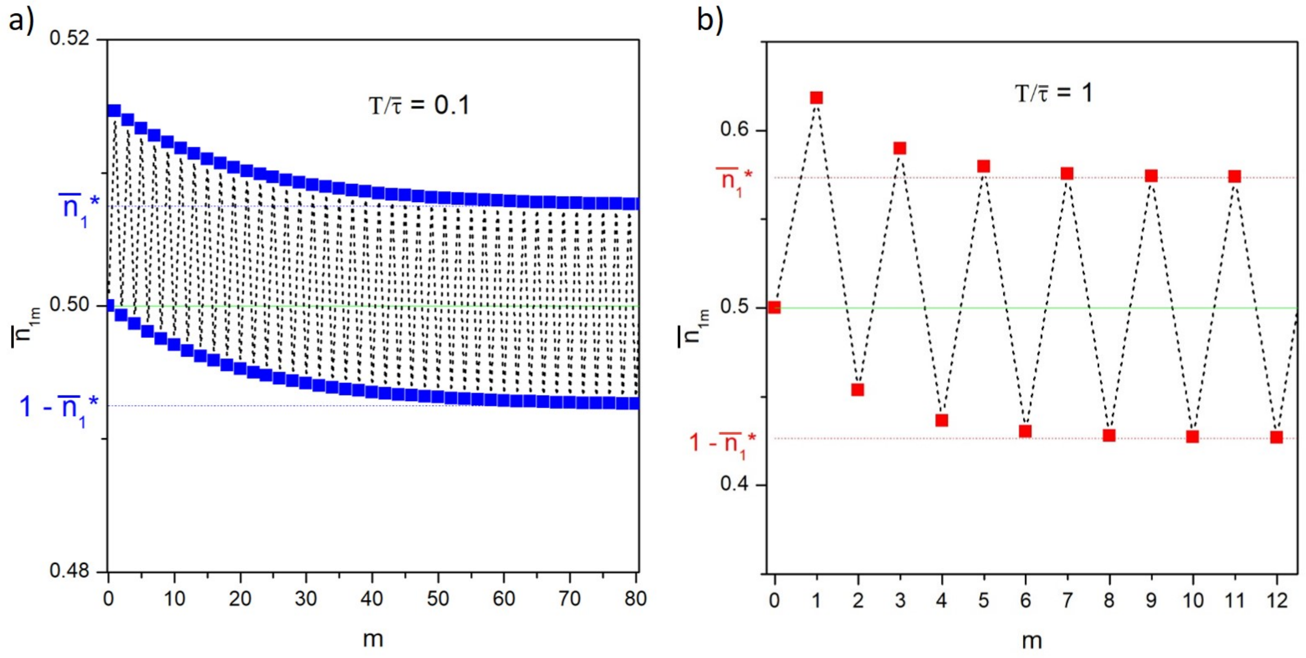

Figure 3. Initial transient of the maximum/minimum values of the reduced occupancy number $\overline{n}_1$ for two values of the ratio $T/\overline{\tau}$; m is the number of half periods since the SW field has been turned on. At the initial time ($m = 0$), $\overline{n}_1 = 1/2$.

$$
\begin{aligned}
P_{2n+1} &= \frac{1}{\pi}\int_0^{2\pi} \overline{M}(x)\cos[(2n+1)x]dx = \\
&= \frac{2}{\pi}\int_0^{\pi} \overline{M}(x)\cos[(2n+1)x]dx \\
Q_{2n+1} &= \frac{1}{\pi}\int_0^{2\pi} \overline{M}(x)\sin[(2n+1)x]dx = \\
&= \frac{2}{\pi}\int_0^{\pi} \overline{M}(x)\sin[(2n+1)x]dx
\end{aligned}
\tag{4}
$$

where the second equality derives from the fact that the integrand has a period of π. Using the explicit expression for $\overline{M}(x)$ over one half period (i.e., the function $\overline{M}^{(A\to B)}(x)$ derived from Equation 15 of the main text with $T/2\overline{\tau} \to \pi/\omega\overline{\tau}$), both integrals are easily calculated. The P_{2n+1}, Q_{2n+1} coefficients turn out to be:

$$
\begin{aligned}
P_{2n+1} &= -\frac{2}{\pi}\left[2\overline{M}_{eq} - M_s(\overline{c}_1 + \overline{c_2})\right]\frac{\omega\overline{\tau}}{1+(2n+1)^2(\omega\overline{\tau})^2} \\
Q_{2n+1} &= -\frac{2}{\pi}\left[2\overline{M}_{eq} - M_s(\overline{c}_1 + \overline{c_2})\right]\frac{(\omega\overline{\tau})^2}{1+(2n+1)^2(\omega\overline{\tau})^2} + \\
&+ \frac{4}{\pi}\overline{M}_{eq}\frac{1}{2n+1}.
\end{aligned}
\tag{5}
$$

The dependence of these coefficients on $\omega\overline{\tau}$ is not unexpected considering that the relaxing magnetization $\overline{M}(t)$ follows an exponential law; the sudden jump of the magnetization at each field inversion produces the second term in the expression for the Q_{2m+1} coefficients. In both limits of very low and very high frequency ($\omega\overline{\tau} << 1$ and $\omega\overline{\tau} >> 1$), the P_{2n+1} coefficients

(associated to the terms in phase quadrature of the Fourier development) vanish for any n, so that the magnetization becomes in phase with the driving field.

The behaviour with frequency of the first three P_{2n+1} and Q_{2n+1} coefficients for $D = 14$ nm as well as of the corresponding magnitudes and phase angles is reported in the Supplementary Information.

References

1. Allia, P., Barrera, G. & Tiberto, P. Hysteresis effects in magnetic nanoparticles: A simplified rate-equation approach. *J. Magn. Magn. Mater.* **496**, 165927 (2020).

2. Valdés, D. P. *et al.* Role of anisotropy, frequency, and interactions in magnetic hyperthermia applications: Noninteracting nanoparticles and linear chain arrangements. *Phys. Rev. Appl.* **15**, 044005 (2021).
